# Effects of diltiazem and metoprolol on levels of high-sensitivity troponin I in patients with permanent atrial fibrillation: a randomized trial

**DOI:** 10.1186/s12872-025-04574-2

**Published:** 2025-03-14

**Authors:** Katrine Enge, Sara Reinvik Ulimoen, Steve Enger, Sophia Onarheim, Mona Olufsen, Are Hugo Pripp, Trude Steinsvik, Christian Hall, Mathias Hetland, Arnljot Tveit

**Affiliations:** 1 Department of Medical Research, Bærum Hospital, Vestre Viken Hospital Trust, Gjettum, Norway; 2 Institute of Clinical Medicine, Faculty of Medicine, University of Oslo, Oslo, Norway; 3 Oslo Centre of Biostatistics and Epidemiology, Oslo University Hospital, Oslo, Norway; 4 Department of Laboratory Medicine, Bærum Hospital, Vestre Viken Hospital Trust, Gjettum, Norway; 5 Department of Medicine, Ringerike Hospital, Vestre Viken Hospital Trust, Hønefoss, Norway; 6 Department of Cardiology, Diakonhjemmet Hospital, Oslo, Norway

**Keywords:** Atrial fibrillation, Biomarkers, Cardiac troponins, Calcium channel blockers, Beta-blockers, Rate control

## Abstract

**Background:**

High-sensitive (hs-) cardiac troponin assays provide prognostic information in atrial fibrillation (AF) patients. Few studies have explored the impact of long-term rate control therapy on levels of troponin in AF patients without coronary heart disease and heart failure. This substudy of the RATe control in Atrial Fibrillation (RATAF) II study aimed to compare the effects of six months’ treatment with diltiazem and metoprolol on hs-troponin I (TnI) levels both at rest and during exercise testing in patients with permanent AF.

**Methods:**

This was a parallel-group, randomized, investigator-blinded clinical trial. The cohort consisted of 93 patients (28 women, mean age 71 ± 7 years) with symptomatic, permanent AF with preserved left ventricular systolic function and no coronary heart disease. Participants were randomized in a 1:1 ratio to receive either diltiazem 360 mg (*n* = 49) or metoprolol 100 mg (*n* = 44) once daily for six months. Blood tests were drawn at rest and during peak exercise testing at baseline, one month and six months’ treatment. This research has been supported by grants from the South-Eastern Norway Regional Health Authority and Vestre Viken Hospital Trust.

**Results:**

Six months’ treatment with diltiazem and metoprolol significantly lowered the heart rate at rest and peak exercise. Both treatment groups exhibited a decrease in hs-TnI levels at rest (diltiazem *p* = 0.008, metoprolol *p* = 0.03) and peak exercise (diltiazem *p* < 0.001, metoprolol *p* = 0.004) at six months compared to baseline levels, with no significant differences observed between the groups.

**Conclusions:**

In patients with permanent AF, six months of rate control therapy with diltiazem or metoprolol lowered levels of hs-TnI. Further research is warranted to determine whether this reduction translates into an improved prognosis.

**Trial registration:**

NCT02695992. Registration date: 2015–04-28.

**Supplementary Information:**

The online version contains supplementary material available at 10.1186/s12872-025-04574-2.

## Introduction

Atrial fibrillation is associated with elevated levels of various biomarkers, some of which hold prognostic significance [1]. Advancements in high-sensitive (hs-) cardiac troponin assays have enabled the detection of lower levels than previously possible, offering valuable prognostic information even in individuals without coronary heart disease. Even minimal rises in troponin levels in the absence of coronary heart disease correlate with poorer prognoses in population-based studies [2–5].

Elevated cardiac troponin levels can predict the onset of atrial fibrillation, [6] and are linked to an increased risk of cardiovascular events and mortality in patients with atrial fibrillation [7, 8]. High-sensitivity assays of troponin I (hs-TnI) and T (hs-TnT) provide robust prognostic data on various clinical outcomes in anticoagulated patients with atrial fibrillation. Troponin levels contribute additional prognostic information regarding stroke, death and major bleeding in atrial fibrillation [7]. These measurements may also augment the prognostic and predictive capabilities of other potent biomarkers [9–13]. The role of biomarkers in atrial fibrillation management is highlighted as an evidence gap in the ESC Guidelines [9, 14].

Previous studies from our group have demonstrated that both calcium channel blockers and beta-blockers significantly reduce hs-TnT and hs-TnI levels in patients with atrial fibrillation after 3 weeks of treatment [15, 16]. However, it is not known whether this effect is sustained over time.

Accordingly, the aim of the current study was to investigate the effects of six months of treatment with the calcium channel blocker diltiazem and the beta-blocker metoprolol on levels of hs-TnI, both at rest and during exercise.

## Methods

### Study design and population

This study was a predefined biomarker substudy of a randomized, single-blinded, prospective parallel-group clinical trial. The effects of six months´ rate control treatment on NT-proBNP levels, rhythm-related symptoms, heart rate and blood pressure have recently been published [17].

We included patients aged 18 years or older with symptomatic permanent atrial fibrillation of at least three months’ duration and a resting heart rate of at least 80 beats per minute (bpm) after discontinuing rate-reducing medication. Patients with left ventricular ejection fraction (EF) below 40% or known coronary heart disease were excluded.

After enrolment and a two-week washout period without any rate-reducing drugs, patients were randomized in a 1:1 ratio to six months of treatment with either diltiazem sustained release capsules 360 mg (Pfizer) or metoprolol slow-release tablets 100 mg (AstraZeneca/Recordati) in a once-daily regimen. The randomization list was computer-generated by the statistician and the hospital pharmacy managed the allocation in accordance with the randomization list. Study personnel and investigators assessing outcomes remained blinded to the allocated drug, whereas participants were not. Blood sampling, clinical assessment and cardiopulmonary exercise test until exhaustion were conducted at baseline (without rate-reducing drugs) and after one and six months. Information on adverse events and symptoms was collected at each study visit, via monthly telephone calls with an experienced study nurse, and through participant-initiated contact as needed.

Patients were recruited from four collaborating hospitals in south-eastern Norway, but all study related procedures took place at the Department of Medical Research, Bærum Hospital. The trial adheres to the recommendations of the Consolidated Standards for Reporting Trials (CONSORT) for randomized, parallel-group trials [18].

## Blood samples and laboratory analyses

Non-fasting venous blood samples for hs-TnI measurement were collected after 30 min of supine rest and at peak exercise. A peripheral intravenous catheter was used for blood collection to minimize patient discomfort. Blood samples for serum analyses were centrifuged at 2000 G for 15 min at room temperature within two hours, then pipetted into aliquots and stored at −80°C.

All blood samples were analyzed in batches to minimize analytical variance. Hs-TnI levels were measured using the Atellica®IM High-Sensitivity Troponin I assay on an Atellica®IM 1600 Analyser (Siemens) with a measuring interval of 2.50–25.000.000 ng/L. Results below the lower limit of detection were assigned the value of 2.5 ng/L. The calculated coefficient of variation for hs-TnI in our laboratory was 9.4% for 21 ng/L, 5.6% for 50 ng/L, 4.2% for 650 ng/L and 3.5% for 7450 ng/L. The assay has a 99th percentile upper reference limit in a healthy population of 45.43 ng/L (men 53.53 ng/L, women 38.64 ng/L).

## Sample size

The sample size was calculated based on the primary outcome results observed in the initial RATAF study [19]. The intent was to obtain a power of 80% with a significance level of 5%, using two-tailed tests. In December 2020, the Steering committee decided to reduce the sample size from 120 patients in each group to 100 in total because of slower inclusion rate and the hit of COVID-19 pandemic. In June 2021, the study was eventually ended prematurely because of slow recruitment.

## Statistical analyses

Continuous variables with a normal distribution are reported as mean ± standard deviation (SD), while non-normally distributed continuous variables are summarized using the median (25th and 75th percentiles). Categorical variables are presented as frequencies (%). The assay’s respective minimum value was used for blood sample values falling below the lower detection limit. Depending on the distribution, bivariate parametric or non-parametric correlation was used to analyse the relationship between continuous clinical variables and outcomes. Continuous variables were compared using the Student’s t-test or the Mann–Whitney U-test, according to data distribution. Comparison of categorical data was done using the Chi-square test or Fisher's exact test. The Wilcoxon signed-rank test was used to compare changes within a treatment group from baseline to a visit or changes from rest to peak exercise. Spearman rank correlation was used to assess the correlation between troponin levels and clinical variables.

To analyze the difference in effect between the two treatment groups, a linear mixed model was employed, applying maximum likelihood estimation. The outcome variable (baseline value) served as a covariate, adjusted for by including it as an independent variable. Fixed effects included treatment time (two points) and group (two categories), as well as the interaction between time and group. An individual-specific random intercept was included to account for observation dependencies within subjects. Logarithmically transformed values of hs-TnI were used for the linear mixed model analyses to account for a right-skewed distribution. All principal analyses were performed using all available randomized patients (intention-to-treat population). A two-tailed p-value below 0.05 was considered statistically significant. All statistical analyses were executed with STATA 17. (StataCorp.2017. Stata Statistical Software: Release 17.0. College Station, TX: Stata-Corp LLC).

## Results

**Fig. 1:**
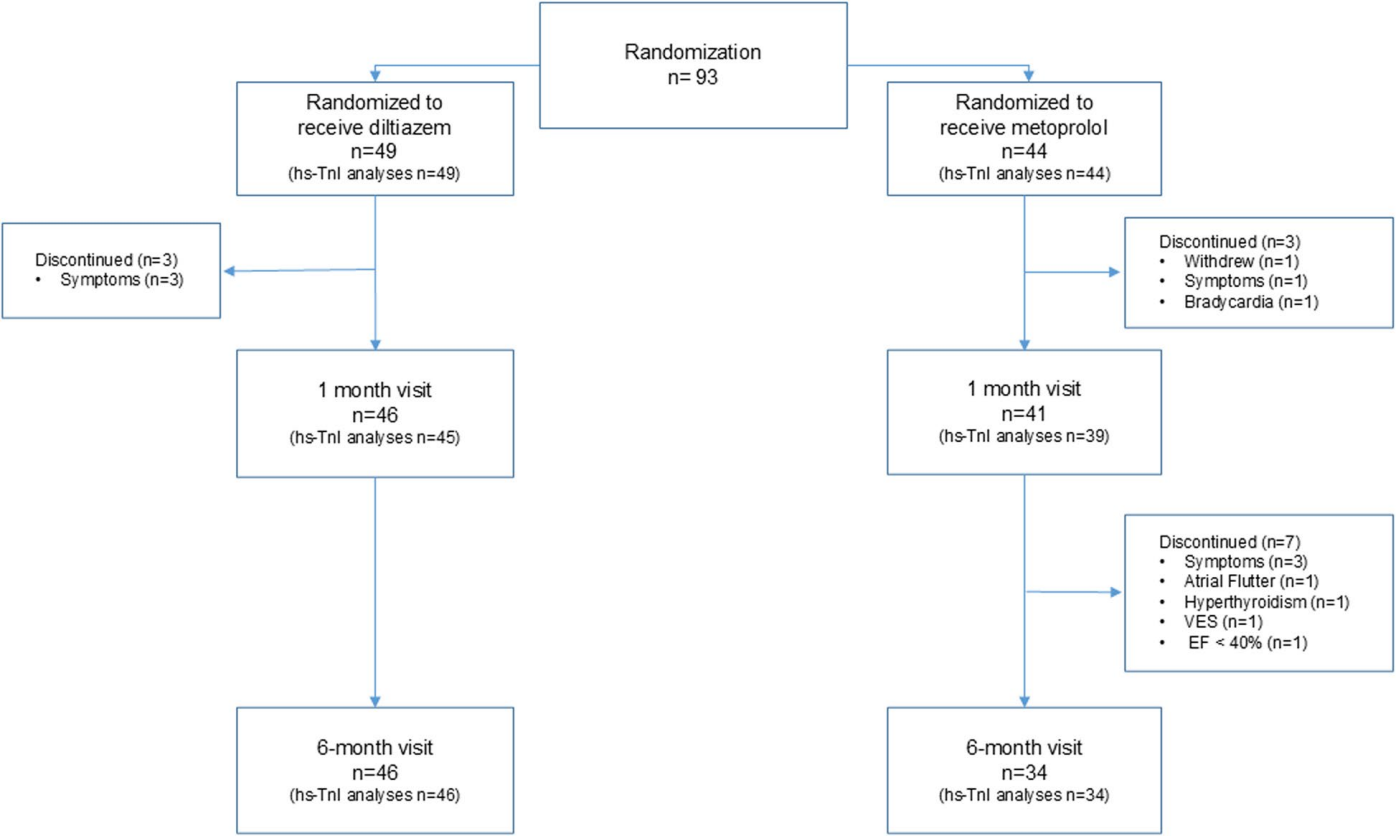
Flow chart of the randomized patients. The number of patients with blood samples available for hs-TnI analyses are presented in parentheses

In total, 93 patients with permanent atrial fibrillation (28 women, 65 men) were randomized to diltiazem (*n* = 49) or metoprolol (*n* = 44) from April 2016 to April 2021. Clinical characteristics are summarized in Table 1. A flow chart of the inclusion process is previously published [17]. The flowchart of the current substudy is illustrated in Fig. 1.

**Table 1:** Baseline characteristics for randomized patients

Variable	Total *n* = 93	Diltiazem *n* = 49	Metoprolol *n* = 44
Age (years)	71.1 (6.7)	70.7 (6.0)	71.5 (7.4)
Sex (women)	30.1% (28)	24.5% (12)	36.4% (16)
CHA_2_DS_2_-VASc score	2.5 (1.4)	2.1 (1.2)	2.9 (1.5)
Low Density Lipoprotein (mmol/L)	3.0 (0.8)	3.2 (0.9)	2.8 (0.7)
Estimated GFR (mL/min)	77.2 (12.3)	76.9 (12.3)	77.6 (12.5)
Haemoglobin (g/dL)	15.1 (1.2)	15.2 (1.2)	14.9 (1.2)
Diabetes type 2	12.9% (12)	4.1% (2)	22.7% (10)
Hypertension	65.6% (61)	57.1% (28)	75.0% (33)
Hypercholesterolemia	39.1% (36)	35.4% (17)	43.2% (19)
Previous stroke/ TIA	17.2% (16)	14.3% (7)	20.5% (9)
Chronic obstructive pulmonary disease	4.3% (4)	2.0% (1)	6.8% (3)
Asthma	10.8% (10)	10.2% (5)	11.4% (5)
Cigarette smoking status			
Current	5.4% (5)	4.1% (2)	6.8% (3)
Former	51.6% (48)	53.1% (26)	50.0% (22)
Never	43.0% (40)	42.9% (21)	43.2% (19)
Body Mass Index (kg/m^2^)	28.9 (4.3)	29.2 (4.2)	28.4 (4.5)
LVEF (%)	57.6 (9.3)	57.2 (8.2)	58.1 (10.5)
Systolic blood pressure (mmHg)	132.8 (14.5)	133.2 (14.0)	132.4 (15.2)
Diastolic blood pressure (mmHg)	88.8 (11.7)	89.2 (12.6)	88.2 (10.7)
Heart rate at rest (bpm)	95.3 (16.0)	94.8 (16.5)	95.9 (15.6)

Values expressed as means ± SD, median (range) or n (%)

CHA_2_DS_2_-VASc score = a score of stroke risk assessment in AF patients, higher score indicating greater risk; Estimated GFR, glomerular filtration rate, estimated from levels of creatinine, age and gender; TIA, transient ischemic attack; LVEF, left ventricular ejection fraction; bpm, beats per minute

### Hs-TnI levels

####  Hs-TnI at rest

**Fig. 2:**
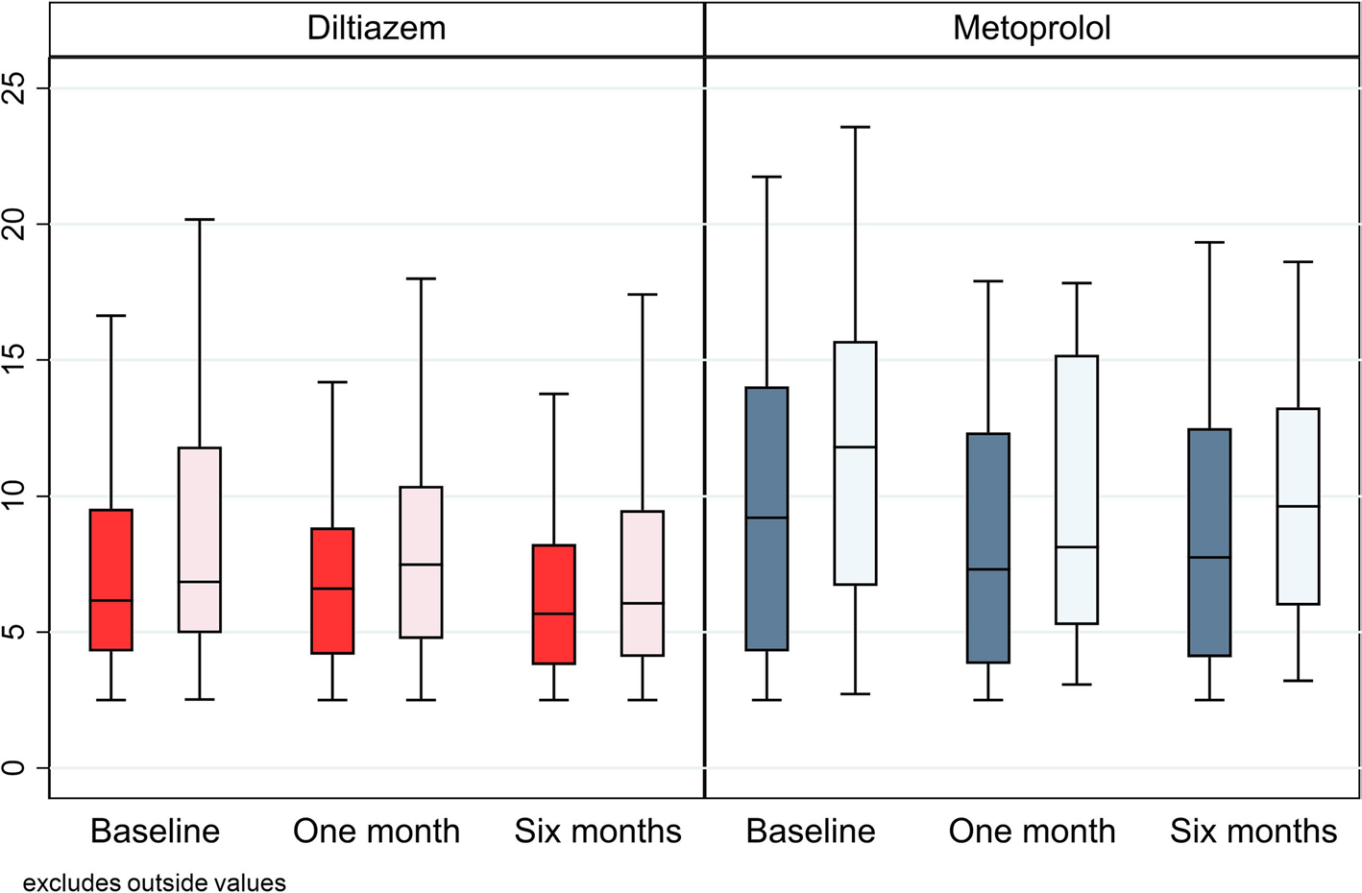
Boxplot of levels of hs-TnI at rest and peak exercise at baseline, one month and six months. Median levels of hs-TnI (ng/L) at rest (dark bars) and peak exercise (light bars) at baseline and follow-up visits at one month and six months. Centre lines show the medians, box limits depict 25th and 75th percentiles. Whiskers encompass 1.5 times the interquartile range from the 25th and 75th percentile

Resting levels of hs-TnI were measurable in 96% of the patients at baseline, with a median level of 7.12 ng/L (4.29, 11.85). In the diltiazem group, levels increased at one month (*p* = 0.03) followed by a significant decrease at six months (*p* = 0.008) compared to baseline. In the metoprolol group, levels decreased significantly at one (*p* < 0.001) and six months (*p* = 0.03) compared to baseline. No significant between-group differences were observed in the change in resting levels of hs-TnI at either one or six months. (Table 2*, *Fig. 2).

**Table 2:** Hs-TnI levels at rest and peak exercise

Study visit	Baseline		1 month		6 months	
Variable	Diltiazem	Metoprolol	Diltiazem	Metoprolol	Diltiazem	Metoprolol
Hs-TnI at rest (ng/L)	6.16 (4.29, 9.54)	9.21 (4.29, 14.03)	6.6 (4.17, 8.85)	7.31 (3.83, 12.33)	5.68 (3.79, 8.24)	7.75 (4.08, 12.49)
Hs-TnI at peak (ng/L)	6.85 (4.96, 11.81)	11.79 (6.7, 15.7)	7.49 (4.75, 10.39)	8.13 (5.26, 15.19)	6.06 (4.09, 9.49)	9.63 (5.98, 13.25)
Hs-TnI Δ^a^ (ng/L)	1.21 (0.65, 2.2)	1.90 (1.23, 2.61)	1.02 (0.78, 1.6)	1.6 (0.92, 2.56)	0.99 (0.33, 1.42)	1.78 (0.89, 2.26)
TnI Δ/rest^b^ (ng/L)	0.18 (0.13, 0.24)	0.20 (0.13, 0.28)	0.17 (0.12, 0.25)	0.25 (0.15, 0.36)	0.14 (0.07, 0.23)	0.22 (0.11, 0.27)

Values are expressed as median (25th percentile, 75th percentile)

^a^Absolute increase from rest to peak = peak levels – resting levels

^b^Relative change from rest to peak = (peak levels – resting levels)/resting levels

#### Hs-TnI at peak exercise

Hs-TnI levels significantly increased from rest to peak exercise in both treatment groups at all visits. There were no between-group differences in the hs-TnI increase from rest to peak at the follow-up visits, although the levels were numerically higher in the Metoprolol group. (*Values are given in *Table 2*, *Fig. 2) Hs-TnI levels at peak exercise were detectable in all patients at baseline, with a median level of 8.38 ng/L (5.17, 14.88). In the diltiazem group, hs-TnI at peak exercise significantly increased at one month (*p* = 0.02), followed by a significant decrease at six months compared to baseline (*p* < 0.001). In the metoprolol group, hs-TnI at peak exercise decreased significantly at one (*p* = 0.003) and six months (*p* = 0.004) compared to baseline. No significant between-group differences in hs-TnI levels at peak exercise were noted at either follow-up visit compared to baseline levels.

#### Sex differences

Resting and peak hs-TnI levels were consistently numerically higher in men, but there was no significant sex difference. There were no sex differences in the increase in hs-TnI levels from rest to peak, although the increase was numerically higher in women at all visits.

#### Left ventricular function

There were no significant differences in baseline levels of hs-TnI at rest or at peak exercise between patients with an EF ≥ 50% compared to those with an EF of 40–49%. No significant between-group differences were noted at either follow-up visit compared to baseline levels.

For more specifics regarding between-group differences, results of correlation between levels of troponins and clinical variables, and an overview of excluded patients and adverse events, see the *Additional file 1*.

## Discussion

Six months of rate control with diltiazem and metoprolol significantly reduced levels of hs-TnI both at rest and during exercise in patients with permanent atrial fibrillation. These findings confirm the results from the initial RATAF study, indicating a stable long-term effect rather than a transient response to treatment initiation [15].

Hs-TnI levels were detectable in nearly all subjects at baseline, despite an EF ≥ 40% and no known coronary heart disease. The troponin levels appear similar to those observed in other permanent or persistent atrial fibrillation populations [7, 15, 16, 20, 21].

Both diltiazem and metoprolol effectively lowered hs-TnI at rest and peak exercise after six months. Notably, in the diltiazem group, baseline levels were low, followed by a *rise* at one month and a subsequent significant decrease at six months. This pattern contrasts with the first RATAF study, where all study drugs lowered troponin levels after three weeks of treatment [15]. The reason behind the initial rise followed by a subsequent decrease in hs-TnI levels in the diltiazem group remains unclear. Further research is needed to understand the underlying mechanism driving this pattern.

Few studies have monitored the course of cardiovascular biomarkers in patients with atrial fibrillation over time, with limited follow-up periods, and diverse populations [20–22].

Similar to the first RATAF study, both diltiazem and metoprolol reduced levels of troponins. Intriguingly, only diltiazem lowered levels of N-terminal prohormone of natriuretic peptide (NT-proBNP) whereas metoprolol increased NT-proBNP levels [15, 17]. This probably reflects different mechanisms behind the release of the different biomarkers.

The exact mechanisms for detectable troponin in atrial fibrillation patients without coronary heart disease or systolic heart failure are not fully understood [7, 10, 23]. Possible mechanisms include myocardial ischemia related to rapid ventricular rate, inflammation, variations in microvascular circulation and myocardial fibrosis [4, 24–28].

Heart rate has been shown to predict TnI levels in atrial fibrillation patients [29]. Despite a baseline mean resting heart rate already below the *lenient* rate of 110 bpm, [30] we observed that a reduction in troponin levels accompanied a further reduction of the heart rate. As reflected in the ESC guidelines, the RACE II data did not find differences between lenient and strict rate control. However, the follow-up time was limited, and we cannot rule out that it still is beneficial with a stricter rate reduction in a longer perspective. Although the reduction of resting heart rate coincided with the lowering of resting hs-TnI, this correlation was not significant in our study.

Previous studies have shown a decrease in troponin levels in hypertensive patients following antihypertensive therapy with amlodipine [31] and combination regimens [32]. In the first RATAF study, there was no correlation between changes in troponin levels and changes in systolic blood pressure [15]. In the present study, systolic blood pressure did not change significantly from baseline to six months, suggesting that changes in blood pressure are unlikely to be the primary mechanism behind the reduction in troponin levels.

Troponin levels are closely linked to the risk of cardiovascular death or stroke in patients with atrial fibrillation [10, 23]. Current guidelines suggest measuring hs-cardiac troponins as part of the supplementary diagnostic work-up and follow-up for selected atrial fibrillation patients [9]. Repeated troponin assessments in a clinical setting could help monitor changes in risk and target management more effectively [33].

Even minor changes in troponin levels can carry important clinical implications and translate to changes in prognosis and risk of clinical events [33]. The sensitivity of troponins suggests that even small reductions observed with rate-reducing treatment may benefit prognosis. In the first RATAF trial, Ulimoen et al. speculated that the reduction in troponin levels observed after three weeks of rate control could improve prognoses over time, by reducing the risk of cardiovascular events [15]. Our confirmation that these findings persist after six months of treatment increases the likelihood of a lasting effect of rate control treatment.

### Limitations

Several limitations should be considered when interpreting the results. Participants were primarily recruited from a defined region in the eastern part of Norway, were all of western origin, and the majority were men. Participants needed to be capable of attending recurrent study visits, which may have led to the exclusion of those with severe mobility issues or other significant health problems. The study included patients with a left ventricular EF ≥ 40% and no known coronary heart disease. However, we did not routinely perform coronary angiography, and therefore cannot exclude the possibility that some of the patients had subclinical coronary heart disease.

## Conclusions

In patients with permanent atrial fibrillation, six months of rate-reducing treatment with diltiazem or metoprolol reduced levels of hs-TnI both at rest and during exercise, with no significant difference between the two treatment groups. Further research is necessary to determine whether the observed reduction in troponin levels due to long-term rate control treatment will lead to an improved prognosis in these patients.

## Supplementary Information

Supplementary Material 1

Supplementary Material 2

## Data Availability

The dataset generated and analyzed during the current study is not publicly available because the Data Protection Authority approval and patient consent do not allow for such publication. However, data are available from the corresponding author on reasonable request.

## Abbreviations

AF: Atrial fibrillation
Bpm: Beats per minute
CHA_2_DS_2_-VASc score: A score of stroke risk assessment in AF patients
EF: Ejection fraction
ESC guidelines: European Society of Cardiology guidelines
Estimated GFR: Estimated glomerular filtration rate
Hs-: High-sensitive
Hs-TnI: High-sensitive assays of troponin I
Hs-TnT: High-sensitive assays of troponin T
LVEF: Left ventricular ejection fraction
NT-proBNP: N-terminal prohormone of natriuretic peptide
RACE II: Rate Control Efficacy in Permanent Atrial Fibrillation II
RATAF: RATe control in Atrial Fibrillation
TIA: Transient Ischemic Attack
TnI: Troponin I
