# Supplementary material for: Effects of diltiazem and metoprolol on levels of high-sensitivity troponin I in patients with permanent atrial fibrillation: a randomized trial
Source: BMC Cardiovasc Disord. 2025 Mar 14;25:181. doi: 10.1186/s12872-025-04574-2 (PMC11907844; doi:10.1186/s12872-025-04574-2)
Supplement: Supplementary file 1 — Supplementary Material 1 [file 12872_2025_4574_MOESM1_ESM.pdf]

Additional Files

Results

Correlations

Hs-TnI at rest

Resting levels of hs-TnI at baseline were moderately correlated to NT-proBNP levels at rest (r_s=0.32; p=0.0015), NT-proBNP levels at peak exercise (r_s=0.32; p=0.0022), age (r_s=0.30; p=0.004), and BMI (r_s=-0.27; p=0.008).

Resting levels of hs-TnI at one month were moderately correlated to NT-proBNP at rest (r_s=0.38; p=0.0004) and at peak exercise (r_s=0.33; p=0.003). Also correlated to resting systolic (r_s=0.28; p=0.013) and diastolic (r_s=0.30; p=0.006) blood pressure, and age (r_s=0.29; p=0.008).

Resting levels of hs-TnI at six months was correlated to NT-proBNP at rest (r_s=0.30; p=0.006) and at peak (r_s=0.28; p=0.016), peak heart rate during exercise (r_s=-0.28; p=0.016), age (r_s=0.30; p=0.008) and BMI (r_s=-0.29; p=0.010).

No statistically significant correlations were found between the relative or absolute change in resting hs-TnI levels versus the relative or absolute change in resting heart rate at six months.

Hs-TnI at peak exercise

Peak levels of hs-TnI at baseline moderately correlated to NT-proBNP at rest (r_s=0.33; p=0.002) and at peak (r_s=0.34; p=0.001), and age (rs=0.28; p=0.01).

Peak levels of hs-TnI at one month was correlated to NT-proBNP at rest (r_s=0.38; p=0.001) and peak (r_s=0.36; p=0.001), diastolic blood pressure (r_s=0.27; p=0.015), age (r_s=0.28; p=0.015) and BMI (r_s=-0.26; p=0.019).

Peak levels of hs-TnI at six months correlated to NT-proBNP at rest (r_s=0.33; p=0.005) and peak (r_s=0.31; p=0.008), resting heart rate (r_s=-0.27; p=0.019), and heart rate at peak exercise (r_s=-0.32; p=0.006).

Sex Differences

When comparing resting hs-TnI levels between sexes (women 5.76 ng/L (3.91,12.49), men 7.74 ng/L (4.44, 11.74)), there was no significant difference.

Adverse Events

After randomization, 13 patients were discontinued: 10 in the metoprolol group and three in the diltiazem group. Each patient experienced, on average, 2.7 adverse events: 2.3 in the diltiazem group and 3.0 in the metoprolol group. There was no significant difference between the two treatment groups. During the course of the study, eight Serious Adverse Events ensued, but none were considered by the safety manager to be associated with the allocated drug.

Table 1: Results from linear mixed model analyses of treatment with metoprolol compared to diltiazem with log transformed values for hs-TnI.

	**Group difference at one month**	**Group difference at six months**
	**Metoprolol vs. Diltiazem**	**Metoprolol vs. Diltiazem**
**Hs-TnI at rest**	-0.099<br>P=0.148<br>(-0.233, 0.035)	0.113<br>P=0.107<br>(-0.025, 0.251)
**Hs-TnI at peak**	-0.052<br>P=0.425<br>(-0.178, 0.075)	0.110<br>P=0.102<br>(-0.022, 0.241)
**Hs-TnI increase during exercise (peak-rest)**	0.044<br>P=0.816<br>(-0.325, 0.413)	0.344<br>P=0.086<br>(-0.049, 0.736)

Values are presented as the between-group difference, P-value and 95% CIs.
